# 4092 TL1 Team Approach to Investigating Host-Microbe Interactions in Cancer Using a 3D Perfusion Culture System

**DOI:** 10.1017/cts.2020.361

**Published:** 2020-07-29

**Authors:** Rachel Chantal Newsome, Derek L Hood, Qin Yu, W. Gregory Sawyer, Christian Jobin

**Affiliations:** 1 University Of Florida Clinical and Translational Science Institute; 2 University of Florida

## Abstract

OBJECTIVES/GOALS: We seek to develop a 3D perfusion culture imaging plate for human fecal bacteria co-culture with epithelial cells in a structure that mimics the gut epithelium. We will develop this system for use with patient fecal samples to characterize patient risk of developing cancer. METHODS/STUDY POPULATION: *E. coli* NC101, a strain that harbors the *pks* gene island, produces the genotoxin colibactin which causes DNA damage that can lead to colorectal cancer development. The genotoxic ability of this bacterium is dependent upon cell-to-cell contact. Here, we present 3D printed *E. coli* NC101 and intestinal epithelial cells (IEC-6) in a perfusion imaging plate, enabling visualization of the cytotoxic effects of the bacteria in real time using confocal microscopy, in combination with flow cytometry analysis for cell cycle arrest (a surrogate marker of DNA damage). RESULTS/ANTICIPATED RESULTS: 40,000 IEC-6 cells were 3D printed in a cylindrical layer in our triple well imaging plate. The cells were infected at an MOI of 100 for 18 hours and time lapse images of the infection were recorded by confocal microscopy. The cells were then harvested for analysis by flow cytometry for cell cycle arrest as a measure of DNA damage. Our images and flow cytometry data show that E. coli NC101 co-localizes with IEC-6 cells and causes cell cycle arrest in phase G2 (infected %G2 = 40.1), compared to uninfected cells (%G2 = 24.7, P = 0.034). Mutant strains lacking adhesion protein FimH or the ability to produce colibactin do not cause G2 cell cycle arrest (P = 0.844 and P = 0.644, respectively). DISCUSSION/SIGNIFICANCE OF IMPACT: We are able to recapitulate the DNA damage phenotype of E. coli NC101 in our 3D culture system. We show here that host-microbe interactions leading to cancer can be modeled in our 3D perfusion system, and we will next use patient fecal samples in our culture system.

